# Longitudinal effects of common carotid artery stenosis on ocular hemodynamics assessed using laser speckle flowgraphy in a rabbit model

**DOI:** 10.1038/s41598-020-72556-9

**Published:** 2020-09-28

**Authors:** Aishah Ismail, Hui Cheng Chen, Ibrahima Faye, Tong Boon Tang

**Affiliations:** 1 Centre for Intelligent Signal and Imaging Research (CISIR), Universiti Teknologi PETRONAS, Bandar Seri Iskandar, Perak Malaysia; 2 Department of Companion Animal Medicine and Surgery, Universiti Putra Malaysia, Serdang, Selangor Malaysia

**Keywords:** Stroke, Optical imaging, Predictive markers

## Abstract

Real-time impairment of ocular blood flow (OBF) under common carotid artery stenosis (CCAS) has not been ascertained. We aimed to longitudinally assess the impact of CCAS on OBF using a rabbit model. About 75% stenosis was created by tying the common carotid artery with a plastic mandrel using a nylon suture. The plastic mandrel was gently removed, leaving a ligature. Neurological and behavioral assessments were recorded as the clinical indicator of stroke severity. With laser speckle flowgraphy, the pulse waveform parameters namely mean blur rate (MBR), blowout score (BOS), blowout time (BOT), rising rate, S1-area, falling rate (FR), S2-area, flow acceleration index (FAI), acceleration time index, resistive index (RI) and the difference between the maximum and minimum values of MBR (AC) were assessed in overall, vessel, and tissue regions of the optic nerve head (ONH). Longitudinally, BOS significantly increased until day 19 post-surgery, whereas FAI, RI, and AC significantly decreased. Beyond day 19, BOS, BOT, FR, FAI, RI, and AC significantly decreased. We defined two stages representing impaired vessel conditions, namely the vessel resistance phase, where BOS increases and FAI, RI, and AC decrease, and the vessel elasticity phase where BOS, BOT, FR, FAI, RI and AC decrease. These stages provide information about atherosclerosis, assessable non-invasively through the eye.

## Introduction

Atherosclerosis or stenosis is one of the major underlying causes of stroke, which asymptomatically reduces blood flow to the brain. It might cause ocular disease because the eyes are an extension of the brain. Generally, the eyes are considered parts of the central nervous system (CNS), as the optic nerve head (ONH) is an extension of the CNS with similar physiological properties of the blood–brain barrier. Impairment of the CNS will cause a cascade of damages to the ONH. Therefore, dysregulation of ocular blood flow (OBF) reflects dysregulation of cerebral blood flow^1^. Studies have shown several ocular impairments resulting from stroke and chronic stenosis: retinal vascular diseases, impaired visual acuity, glaucoma, microaneurysms and retinal hamorrhage^2,3^. Specifically, evidence indicates that OBF dysregulation in the ONH may have an important role as a risk factor for stroke^1,4^. However, the characteristics of OBF dysregulation resulting from atherosclerosis remain largely unexplored, as the longitudinal assessment of patients remains challenging. Hence, understanding the characteristics of OBF dysregulation is essential for early detection and timely disease management.

Small animal models (e.g., mice, rats and rabbits) are indispensable for preclinical investigations, and the selection of an appropriate animal model for atherosclerosis is crucial. In this study, adult New Zealand White (NZW) rabbits were chosen because they have been widely used mainly in stroke^5^ owning to the similar anatomy of their Circle of Willis, which supplies blood from the common carotid artery (CCA) to the brain^6^, to that of humans. Moreover, they are also used in ocular-related studies because of their eye size^7,8^.

Laser speckle flowgraphy (LSFG) is an ocular imaging modality that quantifies OBF in the ONH based on the speckle phenomenon. It has gained favor owning to the reproducibility of its noninvasive measurements in human and animal applications^6–9^. An essential parameter measured by LSFG is the mean blur rate (MBR), which statistically defines the rate of speckle pattern blurring resulting from moving blood cells^9^. Studies have shown that the MBR decreases with age^10^. With aging, atherosclerosis is more likely to occur because vessel elasticity is weakened as a result of impaired vascular repair mechanism, wall injuries, and plaque deposition. Furthermore, associated diseases such as hypertension and diabetes also contribute to the vessel elasticity impairment^11^. Several studies have reported that carotid artery stenosis due to atherosclerosis results in impaired OBF as measured by using the MBR^12,13^. However, the characteristics of ocular hemodynamic remain unknown.

The longitudinal impact of chronic stenosis on ocular hemodynamics has not been investigated thus far. We hypothesized that carotid artery stenosis may cause dysregulation of OBF, and that characterization of OBF dysregulation may provide insights about the vessel condition. In this study, we aimed to understand the characteristics of OBF dysregulation when the right common carotid artery (RCCA) is occluded, and to assess the potential of OBF dysregulation as a marker of impairment in cerebral blood flow. We used neurological and behavioral deficits as a reference measure of RCCA stenosis (RCCAS) and clarified the relationship between ocular hemodynamics and neurological behavior. To our best knowledge, this is the first longitudinal in vivo study on the impact of OBF pulse waveform (PWF) parameters during RCCAS in a rabbit model.

## Results

### Neurological and behavioural assessment (NBA)

At 24 h postoperatively, all rabbits showed recovery by reaching out for food and water, although with much less active, indicating successful surgical procedures. The rabbits survived throughout the study with no infection or sickness, suggesting the absence of postoperative complications. NBA scores as the indicator of RCCAS severity were analyzed using one-way repeated measure analysis of variance (ANOVA), which showed a significant change (*p* < 0.001) in scores over time, indicating neurological and behavioral deficits. The correlation coefficients between the NBA score and the PWF in each region, are summarized in Table 1.

**Table 1:** Summary of correlation coefficients (r) from Spearman correlation coefficient analysis between NBA and PWF parameters in each respective region.

PWF parameters	MA	MV	MT
	r	r	r
MBR	–	− 0.251^⁂^	− 0.225^⁂^
BOS	0.379^⁂^	0.326^⁂^	0.381^⁂^
BOT	− 0.090^⁑^	–	− 0.065^⁂^
RR	− 0.084^⁂^	–	− 0.065*
FR	− 0.068*	− 0.067^⁑^	–
FAI	− 0.310^⁂^	− 0.300^⁂^	− 0.425^⁂^
ATI	–	–	–
RI	− 0.345^⁂^	− 0.345^⁂^	− 0.347^⁂^
AC	–	–	–

*p < 0.05, ^⁑^*p* < 0.01, ^⁂^*p* < 0.001.

### Longitudinal changes of ocular PWF parameters

**Figure 1:**
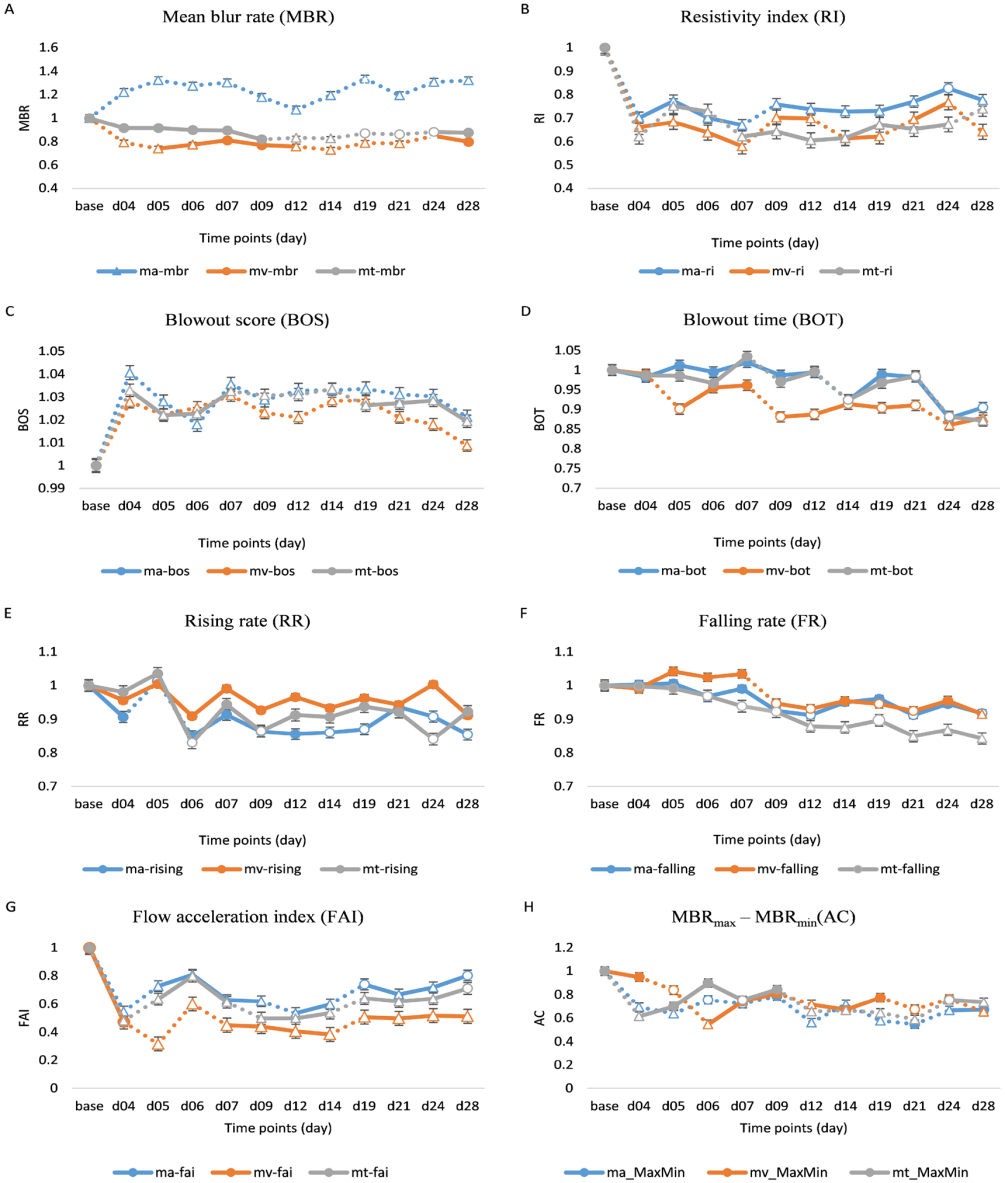
(**A**–**H**). Longitudinal changes in ocular pulse waveform (PWF) parameters. The line graph represents the PWF parameters, (**A**) MBR, (**B**) RI, (**C**) BOS, (**D**) BOT, (**E**) RR (**F**) FR, (**G**) FAI, and (**H**) AC for each region. Vertical bars represent the standard error of means. Results from one-way repeated measures ANOVA are displayed in the line graphs; dotted lines represent a significant difference between each time point. The circle and triangle symbols represent statistically significant differences the between baseline reading and the reading at each respective time point (empty circles: *p* < 0.05 and empty triangle: *p* < 0.001).

Eight baseline readings were taken at random times before RCCAS, and the PWF parameters were analyzed for each region (MA: overall, MV: vessel, MT: tissue). No significant difference was observed between each of the baseline readings at each PWF of each region. The ocular PWF parameters were analyzed based on the three predefined regions, and the PWF parameters that showed significant responses were MBR, maximum value of MBR (MBR_max_), minimum value of MBR (MBR_min_), difference between MBR_max_ and MBR_min_ (AC), blowout score (BOS), blowout time (BOT), rising rate (RR), falling rate (FR), flow acceleration index (FAI), acceleration time index (ATI) and resistivity index (RI). The longitudinal changes in ocular PWF parameters are presented as line graphs in Fig. 1A–H. The MBR responded significantly with *p* < 0.001 in all three regions. MBR-MA (*p* < 0.001) showed a significant increase after RCCAS and remained high until day 28, whereas MBR-MV and MBR-MT showed a significant decrease (*p* < 0.01) after day 12 and day 9 respectively. Both MBR_max_ (MA, MV, MT) and MBR_min_ (MA, MV, MT) significantly increased (*p* < 0.001), whereas AC (MA, MV, MT) significantly decreased (*p* < 0.001) over time. BOS (MA, MV, MT) showed a significant increase (*p* < 0.001) and remained high until day 19 but significantly decreased thereafter. BOT (MA, MV, MT) significantly decreased (*p* < 0.001) (MV: day 4, MT: day 7, and MA: day 12) and FR (MV, MT) (*p* < 0.001) also significantly decreased after day 6. RR-MA (*p* = 0.048), RR-MT (*p* = 0.025), FAI (MA, MV, MT) and RI (MA, MV, MT) all showed a constant decrease over time (all *p* < 0.001).

**Figure 2:**
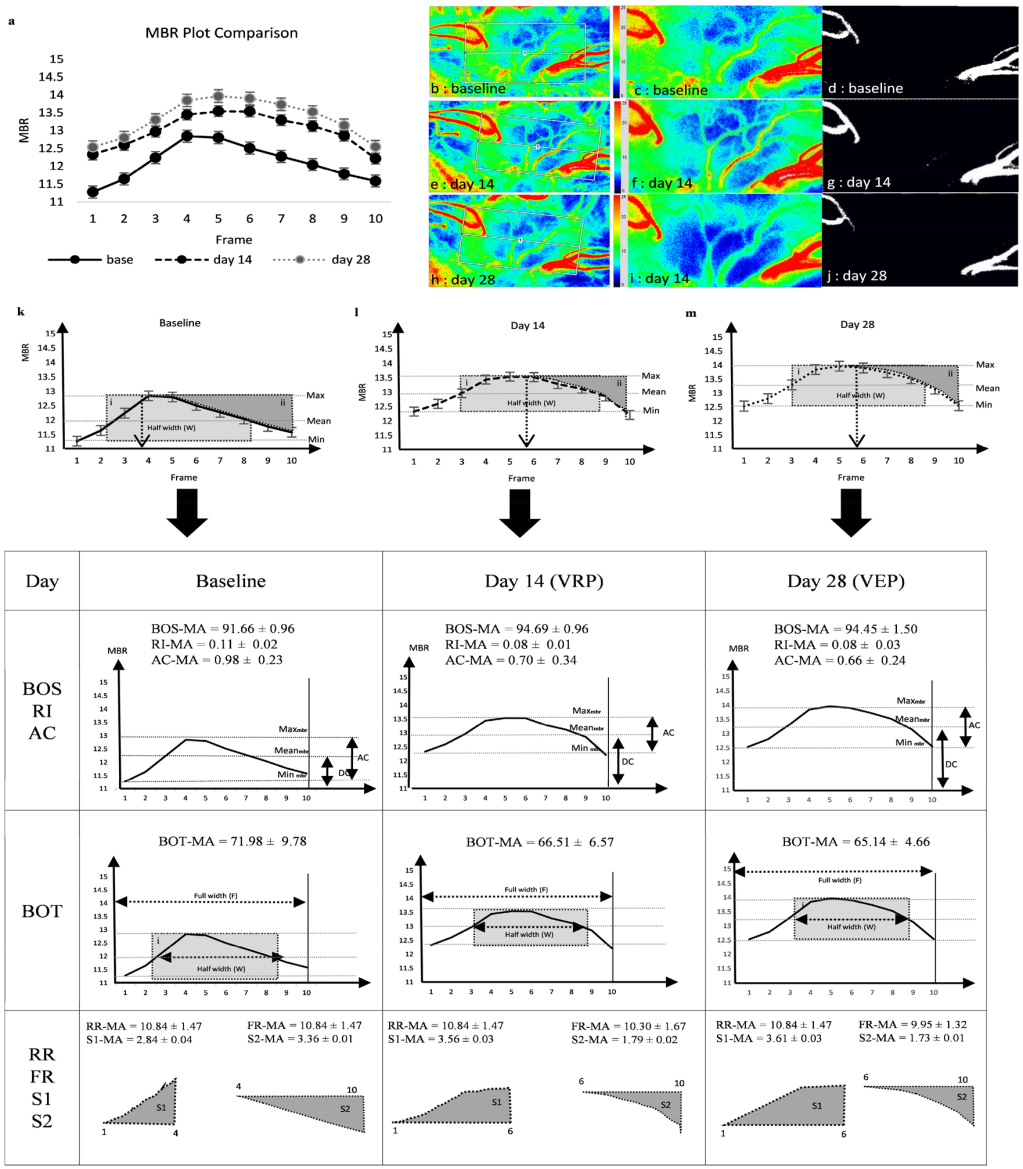
Differences of heartbeat MBR plot between normal, day 14 (VRP) and day 28 (VEP). (**a**) Comparison of synchronized heartbeat MBR plot at baseline, day 14 and day 28. The MBR data were averaged from all rabbits and plotted for each respective time point of one cardiac cycle to permit comparison, vertical bar represents standard error of means. (**b**–**j**) LSFG composite map of the ONH. (**b**,**e**,**h**) The ONH region of interest was selected from the whole frame. (**c**,**f**,**i**) showing ONH region of interest. (**d**,**g**,**j**) vessel (white) and tissue (black) area within the rubber band were segmented out. (**k**–**m**) Comparison of each MBR plot at baseline (**k**), day 14 (**l**) and day 28 (**m**) individually, highlighting the waveform dynamic differences representing VRP and VEP respectively.

Consequently, the raw MBR synchronized heartbeat data of the MA region were averaged from all rabbits and plotted to compare the differences in waveform characteristics between baseline, day 14, and day 28 in one cardiac cycle, as shown in Fig. 2. MBR_max_ and MBR_nin_ showed higher plot (*p* < 0.001) but AC became smaller (*p* < 0.001), and the half width (W) of the blood flow wave, represented as BOT became smaller from baseline to day 28, (all *p* < 0.001). The S1-area became larger, whereas the S2-area became smaller from baseline to day 28 (all *p* < 0.05).The PWF data for each region are summarized in supplementary tables (Table S1: MA, Table S2: MV and Table S3: MV), and the *p* values for AC, S1-area, and S2-area for each region are provided in Table S4.

### Correlation between PWF parameters and NBA score

The PWF parameters that significantly positively correlated with the NBA were BOS-MA and BOS-MT, whereas the PWF parameters that significantly negatively correlated with the NBA score were MBR-MV, MBR-MT, BOT-MA, BOT-MT, FR-MA, FR-MV, FAI-MA, FAI-MV, FAI-MT, RI-MA, RI-MV, and RI-MT (Table 1).

Multivariate regression analysis was applied to identify the PWF parameters that influenced the NBA score. MBR, BOS, BOT, RR, FAI, ATI, and RI were statistically significantly associated with the NBA in the MA [*F*(8, 1,306) = 32.393, *p* < 0.0004], MV [*F*(8, 1,453) = 32.393, *p* < 0.0004] and MT [*F*(8, 1,304) = 32.393, *p* < 0.0003] regions. Multivariate regression analysis showed that the PWF parameters associated with the NBA score were MBR (MA, MV), BOS (MA, MV and MT), BOT (MA, MV and MT), RR (MA, MV, and MT), FAI (MA, MT), ATI (MA, MV), and RI (MV) (Table 2).

**Table 2:** Summary of multiple regression analysis of the correlation between each PWF parameters and NBA score in the MA, MV and MT regions.

	MA (R = 0.422)			MV (R = 0. 436)			MT (R = 0.416)		
	B	SE_B_	β	B	SE_B_	β	B	SE_B_	β
MBR	0.504	0.132	0.177^⁂^	0.176	0.044	0.204^⁂^	0.588	0.245	0.134*
BOS	–	–	–	–	–	–	–	–	–
BOT	− 0.214	0.033	− 0.248^⁂^	− 0.210	0.033	− 0.249^⁂^	− 0.201	0.035	− 0.262^⁂^
RR	–	–	–	–	–	–	–	–	–
FR	− 0.882	0.175	− 0.190^⁂^	− 0.957	0.187	− 0.194^⁂^	− 0.812	0.193	− 0.192^⁂^
FAI	–	–	–	− 3.875	0.714	− 0.377^⁂^	–	–	–
ATI	− 0.055	0.025	− 0.113*	–	–	–	− 0.058	0.025	− 0.131*
RI									
AC	− 5.255	0.979	− 0.197^⁂^	− 1.674	0.343	− 0.179^⁂^	− 6.146	1.340	− 0.203^⁂^

R = multiple correlation coefficient, B = unstandardized regression coefficient, SE_B_ = standard error of the coefficient; β = standardized regression coefficient.

*p < 0.05, ^⁑^*p* < 0.001, ^⁂^*p* < 0.0001.

## Discussion

This study provides detailed direct evidence on the longitudinal impact of RCCAS on ocular hemodynamics. MBR waveform, the quantitative parameter that denotes erythrocyte movement in the ONH^14^, reflects the ophthalmic status. In this study, we found that the MBR in the overall region initially demonstrated a significant increase, and subsequently remained high over time, whereas MBR-MV and MBR-MT showed a significant decrease. Aizawa et al.^7^ reported a significant reduction in the MBR tissue region of induced chronic ischemia on the fourth week in a rabbit model. In addition, a case-study about an obstruction in the left internal carotid artery with symptoms of motor weakness and blurred vision in the left eye reported a decrease in the MBR over time^15^. Moreover, the MBR was reported to be lower in diabetes patients^16^ and diabetic rabbits^17,18^. Likewise, the MBR in the vessel and tissue regions was significantly lower in hyperlipidemia patients^19^. Studies have shown that MBR-MT was stable regardless of age^10^ and did not significantly affect in the eyes with the macula^20^. These are because MV consists of large vessels, whereas MT represents small capillaries and the choroidal area. MBR-MT which represents the choroid or deep region of the ONH near the lamina cribrosa^21^ which is partially autoregulated, is controlled by the sympathetic nervous system^22^.

We theorized that the variables of MBR waveform provide an indication of the systemic fluctuation of blood flow. A study showed that, in tissue regions, MBR_max_-increases while MBR_min_ decreases with aging^23^, however, AC was not mentioned. In this study, we observed that the MBR_max_ and MBR_min_ increased while AC decreased in all regions over time. The increase in the MBR was caused by increases in MBR_max_ and MBR_min_, however, the decrease in AC resulted in a flatter waveform, suggesting that the blood flow remained high owning to reduced fluctuation, which indicated a reduction in vessel elasticity. Thereafter, several studies reported that BOS and BOT decreased with aging^26,27^. Notably, study on the correlation between atherosclerosis, age, and OBF found that, with aging, BOT decreased with an increase in brachial-ankle pulse wave velocity (index of arterial stiffness) and a decrease in intima-media thickness (index of atherosclerosis severity) owning to reduced vessel elasticity^24^. In this study, BOS significantly increased and remained high in all regions, indicating that the vessel is in the vessel resistance phase to maintain sufficient blood flow. After day 19 both BOS and BOT started to decrease in all region, demonstrating the reduction of vessel elasticity to maintain the blood flow.

**Figure 3:**
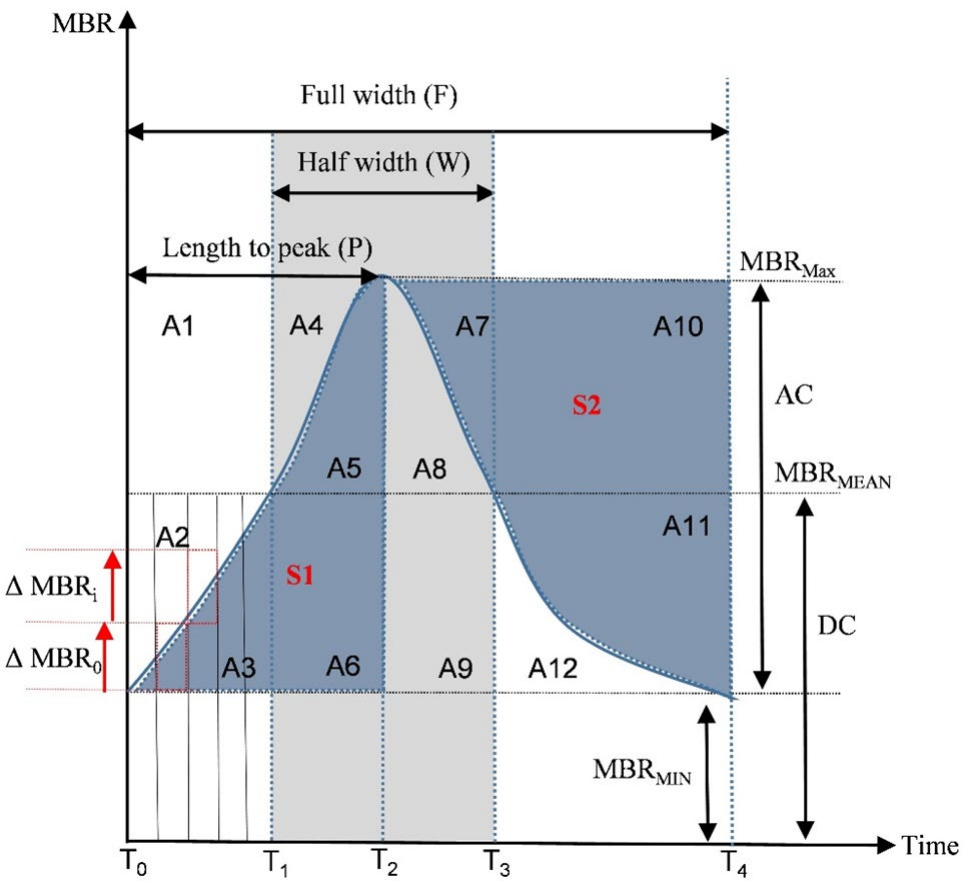
(A1–A12). Schematic explanations of the MBR waveform subdivision area. Area A1 to area A12 are regions representing each PWF parameter.

RI demonstrated a significant decrease over time in all regions. An open-angle glaucoma study, in a young age group reported that, BOS increased with a decrease in RI, suggesting maintenance of stable blood flow^25^. As shown by the waveform area illustrated in Fig. 3, higher BOS with lower RI and AC resulted in a flatter waveform. This study also showed a strong inverse relationship between BOS and RI, in which an increase in BOS with a decrease in RI until day 14 indicated a vessel resistance phase (VRP) to maintain blood flow.

Both RR and FR are high when blood flow increases, and both indices are low when blood flow gently decreases. FR-MT was found to be positively correlated with systolic blood pressure (SBP) and pulse pressure^26^. In hypertension, RR is associated with SBP, and FR is associated with diastolic blood pressure (DBP). High SBP and DBP are the indicators for hypertension, and elderly individuals with high SBP and low DBP are at greater risk for stroke^27^. Considering the aforementioned findings, both RR (MA and MT) and FR, particularly in tissue regions significantly decreased, which indicates a slower decrease of blood flow after the slope, over time in this study. Our findings also showed that from baseline to day 14 and day 28, S1-area increased while S2-area decreased demonstrating that it takes a longer time to reach MBR_max_ and a shorter time to reach MBR_min_ as a result of RCCAS.

We next addressed the effect of RCCAS on neurological and behavioural parameters and further inspect the relationship between the NBA score with the PWF parameters. This study is the first to implement the CCA ligation technique on a rabbit model. Previously, this technique has been limited to intimal hyperplasia studies^28^ in murine models, providing constant ~ 75% closure throughout the study. There have been numerous approaches in inducing stenosis, including injury methods^29,30^ and ameroid constrictor implantations^30,31^. However, these techniques are time consuming and yield inconsistent closure rates, leading to large variations among rabbits^31,32^. In this study, the NBA score was used for a complete behavior characterization to evaluate the impact of ~ 75% RCCA ligation on the brain, as the neurological assessment scale(NAS) and behavioral tests have been practically used separately as a clinical stroke indicator and for rating the stroke severity^33^, pain, and wellness in rabbits^34^. The NBA score revealed clear neurological deficits and behavioral changes with late responses, ears flattened down, less alertness, and less grooming. The experimental results of this study demonstrated the impact of chronic RCCAS on ocular hemodynamics, which, at the same time, indicated neurological symptoms. The multivariate analysis shed light on the relationship and showed that the PWF parameters BOS (MA, MV, MT) significantly positively correlated with the NBA score, whereas MBR (MA, MT), BOT (MA, MT), RR (MT), FR (MA, MV), FAI (MA, MV, MT), and RI (MA, MV, MT) significantly negatively correlated with the NBA score. Taken altogether, in the first phase, BOS was observed to increase while there were continuous decreases in FAI, RI, and AC with no changes in BOT and FR. These results demonstrated a VRP to maintain the perfusion of blood flow. Thereafter, after day 19, BOS, BOT, FR, FAI, RI, and AC significantly decreased, which indicated a transition from the VRP to the vessel elasticity phase (VEP). As a novel finding using ocular PWF parameters, we propose two stages of impaired ocular vessel condition due to chronic stenosis, namely VRP, during which BOS increases with a decrease in FAI, RI, and AC, followed by VEP, during which BOS, BOT, FR, FAI, RI, and AC decrease.

This study has several limitations. The PWF readings from the eyes of rabbits cannot be directly compared to human readings, as rabbits have a higher rate of heartbeat. A higher pulse rate leads to lower *N* frames. Moreover, atherosclerosis in humans has diverse causes and manifestations, whereas stenosis is controllable and highly reproducible in experimental studies, allowing precise investigations, analysis and outcomes. Hence, this study provides insights on how chronic stenosis significantly affects the blood flow to the brain and subsequently to the eyes. Further, the use of a small number of animals was considered sufficient to draw meaningful outcomes in this preliminary study. In addition, *p* < 0.05 was adopted to ensure repeatability^35^. This study showed several PWF parameters had a *p* value of < 0.05, and further demonstrated a highly significant effect (*p* < 0.001).

In summary, the findings of this study confirmed that the CCA stenosis significantly affects ocular hemodynamics, with neurological and behavioral manifestations. Therefore, according to the OBF PWF parameters, we defined two stages, namely VRP and VEP, that describe the severity of the vessel condition based on ocular hemodynamics impairment. These impairment stages are a promising noninvasive tool for discriminating normal from severe risk that signals a stroke event through the eyes. This is the first longitudinal study to measure OBF PWF parameters, in conscious rabbits without any influence of drugs or anesthesia. Further investigations on the impairment of OBF due to atherosclerosis are essential to aid in the detection of the disease and, importantly, to provide a method for early stroke detection.

## Methods

### Animals

In this study, male NZW rabbits weighing 2.5–3.5 kg (n = 10) were used. The sample size was calculated on the basis of a pilot study using power analysis, specifically for small animals in neuroscience and behavioral studies^36^. Smaller groups sizes were favored to make the best use of each rabbit. This study was approved by the Institutional Animal Care and Use Committee of the National University of Malaysia, UKM (UKMAEC:20-MAR./998-2019), and was conducted according to the code of practice for the care and use of animals for scientific purposes. The rabbits were provided with ad libitum food and water, and individually housed under a common enriched environment one month before surgery.

### Surgery of RCCAS

Surgery was performed in a sterile, controlled environment with a room temperature of between 22 and 24 °C. The rabbits were anesthetised with ketamine (35 mg/kg) for muscle relaxation and supplied with 2.0% isoflurane using a face mask for deep anesthesia, with the body temperatures maintained at 37℃ with a heating pad. The neck was cleaned of fur, and swabbed with iodine, and lidocaine was injected under the skin for numbing. A 5 to10 mm skin incision was made to carefully expose the RCCA. The CCA of NZW rabbits has an average diameter of ~ 2 mm^37,38^. Before performing the ligation, the CCA diameter was further confirmed by placing a sterile nylon thread around the CCA, which was then marked using a marker. The CCA diameter of the rabbits was at ~ 2 mm. About 75% stenosis was created by tying the CCA with a plastic mandrel (outer diameter 1.5 mm) with a 9-0 nylon suture^28^, just below the carotid bifurcation. The plastic mandrel was thereafter gently removed, leaving a ligature with 0.5 mm of the CCA diameter (25% of the blood flow diameter). Thereafter, the rabbits were allowed to rest for recovery and were monitored daily for infection. All rabbits were euthanized at the endpoint of the study, or earlier if they showed signs of immobility, extreme discomfort or inability to reach food/water during the study.

### Longitudinal study: measurement of OBF using LSFG

Instead of using typical wooden restraints with the head out, the rabbits were comfortably wrapped in a towel with the paws inside, and the ears free and slightly loose around the neck to keep the animals warm, feeling safe, and comfortable during handling. LSFG measurements were taken from the right eye throughout the study. The baseline readings were randomly taken within one month before surgery, followed by taking readings after the surgery until day 28. The ONH images were captured and the integrated using the LSFG Analyzer software version 3.2.3.0 (Softcare Co, Fukuoka, Japan), which automatically synchronizes the captured MBR images with the beginning and end of the cardiac cycle recorded within 4 s of acquisition time. The synchronized cardiac cycle images were normalized to one image sequence representing a complete cardiac cycle of blood flow in the ONH. A rectangular rubber band was manually set on the ONH region of interest. The same rubber band, at an identical position, was used for the subsequent images of the same rabbit. By using the vessel segmentation function, PWF parameters in the MA, MV, and MT regions were obtained, as presented in Fig. 2b–j. The PWF parameters assessed using LSFG, namely MBR_max_, MBR_min_, AC, BOS, BOT, RR, FR, FAI, ATI, and RI in all three regions were computed. Figure 3 describes the waveform subdivision area of an average MBR graph plotted against time. The waveform is divided into 12 sections (A1–A12). The blue shaded area represents the S1-area (A3, A5, A6) and S2-area (A7, A10, A11), and the gray-shaded area represent the half width of a complete blood flow wave^9^.

BOS, an index for vessel resistance, indicates how much blood flow is being maintained in the vessel, with (DC) denotes the mean quantity of blood flow, and is defined as

1 $$ {\text{BOS}} = \left[ {2{-}\left( {{\text{AC}}/{\text{DC}}} \right)} \right] \times 100 $$

BOT represents the length of time blood flow is maintained at a high level in each heartbeat, signifying the vessel elasticity. The (W) is the half width of the blood flow wave, and (F) is the full width of the blood flow wave and BOT is computed as

2 $$ {\text{BOT}} = 100 \times \left( {{\text{W}}/{\text{F}}} \right) $$

RR quantifies the upward area of the wave, whereas FR quantifies the downward area of the wave, with C set as 25 to match the color balance used in the LSFG analyzing software and computed as

3 $$ {\text{RR}} = {\text{C}} \times {\text{S}}1/Sall_{RR} \;{\text{or}}\;{\text{RR}} = 25 \times \left[ {\frac{{\left( {{\text{A}}3 + {\text{A}}5 + {\text{A}}6} \right)}}{{\left( {{\text{A}}1 + {\text{A}}2 + {\text{A}}4} \right)}}} \right] $$

4 $$ {\text{FR}} = {\text{C}} \times {\text{S}}2/Sall_{FR} \;{\text{or}}\;{\text{FR}} = 25 \times \left[ {\frac{{\left( {{\text{A}}7 + {\text{A}}10 + {\text{A}}11} \right)}}{{\left( {{\text{A}}8 + {\text{A}}9 + {\text{A}}12} \right)}}} \right] $$

FAI indicates the index of the maximum amount of change in MBR, and RI shows the difference of the maximum and minimum MBR by the maximum MBR, and computed as

5 $$ {\text{FAI}} = {\text{Max}}\left( {\Delta {\text{MBRi}}} \right) $$

6 $$ {\text{RI}} = ({\text{MBR}}_{\max } {-}{\text{MBR}}_{\min } )/{\text{MBR}}_{\max } \;{\text{or}}\;{\text{RI}} = {\text{AC}}/{\text{MBR}}_{\max } $$

Thereafter, S1-area and S2-area were compared among baseline, day 14, and day 28 using one-way repeated measure ANOVA. To calculate the area, a second-order trend line was fitted on the MBR heart beat plotted graph to obtain the line regression equation, and the constants *a*, *b*, and *c* [Eq. (9)] were individually computed to obtain the exact curve for the area calculation. Referring to Fig. 3, the S1-area and S2-area were calculated as

7 $$ {\text{S}}1 = \mathop \smallint \limits_{T0}^{T2} y\left( t \right)dt $$

8 $$ S2 = \frac{{\left[ {\mathop \smallint \nolimits_{T2}^{T4} y\left( t \right)dt} \right] \times {\text{FR}}}}{25} $$

9 $$ {\text{where}}\;{\text{y}} = at^{2} + {\text{b}}t + c. $$

### NBA

In this study, the NAS^39^ and behavioral or wellness score^34^ were combined and named as NBA. The NBA was used to evaluate the severity of RCCAS throughout the study. The behavior of each rabbit was examined 24 h after surgery, allowing adequate anesthesia recovery. For NAS, the rabbits were placed on a smooth rubber mat on a flat surface for the evaluation procedure. The NAS score is a clinical indicator of stroke severity in rabbits, without prior stimulus training. The NAS consist of righting reflex, paw (forepaws and hind limbs) dysfunction, and postural reflex. The scoring method is described in Table 3^39^.

**Table 3:** NBA scores as clinical criteria for rating the severity of CCAS.

Test	Clinical criteria	Observation scoring	Potential score
a. NAS	Neck twist	0: normal, 1: twist of the neck	1
	Righting reflex dysfunction	0: normal; righted within 1 s 1: moderate; righted within 5 s 2: severe; did not right within 5 s	2
	Forepaws extension	0: normal; extended within 1 s 1: moderate; extended within 5 s 2: severe; did not extend within 5 s	2
	Hind-limbs extension	0: normal; extended within 1 s 1: moderate; extended within 5 s 2: severe; did not extend within 5 s	2
	Postural reflex	0: normal resistance within 1 s 1: moderate resistance within 5 s 2: severe fell on the contralateral side	2
b. Wellness	Facial grimace scale	0:eyes wide, whiskers downward, nose U shaped 1: eyes slight narrow, whiskers flattened, nose V shaped 2: eyes tightened or closed, whiskers and cheeks flat, nose V shaped, head tucked against the chest	2
	Ears	0: alert, 1: droppy, 2: flattened	2
	Exploration	0: normal, 1: moderate, 2: severe	2
	Eating	0: normal, 1: less eating	1
	Drinking	0: normal, 1: less drinking	1
	Grooming	0: normal, 1: moderate, 2: severe	2
		Total	19

*0, normal; 1, moderate or reduced; 2, severe or absent. A total score of 20 is equivalent to death.

The wellness or behavioral score was used as a post-procedural observation to evaluate pain and stress in the rabbits and was ranked using a numerical scale. The wellness score comprises the facial grimace scale, ears, exploration, eating, drinking and grooming, and the scoring method is described in Table 3. The behavioral assessment was performed three times daily. All scores were summed and represented as the functional score, with 19 as the maximum score, and 20 set as the score for death.

### Statistical analysis

Statistical analysis was performed using SPSS statistical software version 23 (IBM, Armonk, NY). Data on PWF parameters for each region are presented as mean ± standard deviation. The data were observed to follow a normal distribution and outliers were removed. Three-way repeated measure ANOVA was performed to determine significant differences among the three regions (MA, MV and MT) at different time points, which were subsequently confirmed using one-way repeated measure ANOVA (or Friedman’s test when normality is violated) for each region. The values of PWF parameters for each region were averaged and normalized according to the baseline reading and plotted across time. Pearson’s correlation (or Spearman’s rank correlation when normality is violated) analysis was performed to measure the strength and direction of the relationship between the NBA score and PWF parameters. Univariate regression and multivariate regression were applied to identify PWF parameters that had a strong association with the NBA score. In this study, *p* < 0.05 was set as the significance level.

## Supplementary information

Supplementary Information.
