# Supplementary material for: Longitudinal effects of common carotid artery stenosis on ocular hemodynamics assessed using laser speckle flowgraphy in a rabbit model
Source: Sci Rep. 2020 Sep 28;10:15829. doi: 10.1038/s41598-020-72556-9 (PMC7522272; doi:10.1038/s41598-020-72556-9)
Supplement: Supplementary file 1 — Supplementary Information. [file 41598_2020_72556_MOESM1_ESM.docx]

**Longitudinal Effects of Common Carotid Artery Stenosis on Ocular Hemodynamics Assessed Using Laser Speckle Flowgraphy in a Rabbit Model**

Aishah Ismail^1^, Chen Hui Cheng^2^, Ibrahima Faye^1^ and Tong Boon Tang^1*^

Supplementary Table S1: Mean scores of pulse waveform parameters for overall region (mean ± standard deviation) in overall region (MA).

| MA | MBR | BOS | BOT | RR | FR | FAI | ATI | RI | MBR_max_ | MBR_min_ | AC |
| --- | --- | --- | --- | --- | --- | --- | --- | --- | --- | --- | --- |
| *P* value | *** | *** | *** | * | *** | *** | 0.004 | *** | *** | *** | *** |
| base | 10.858  ± 2.235 | 91.659 ±  .957 | 71.983 ±  9.781 | 8.953 ±  2.158 | 10.835 ±  1.471 | .9118 ±  .230 | 44.774 ±  2.711 | .107 ±  .023 | 9.7119 ± 0.686 | 9.320 ± 1.721 | .9792 ± .23481 |
| day04 | 13.247b±  2.006 | 95.387 ±  1.161 | 70.696 ±  12.124 | 8.122 ±  3.999 | 10.872 ±  2.309 | .498 ±  .195 | 50.545 ±  14.221 | .075 ±  .029 | 13.8952 ± 1.898 | 14.384 ±3.419 | .6792 ±.345 |
| day05 | 14.343 ±  2.676 | 94.221 ±  1.273 | 72.872 ±  13.244 | 9.072 ±  3.519 | 10.903 ±  2.132 | .662 ±  .232 | 51.676 ±  21.185 | .083 ±  .027 | 13.0000 ± 3.742 | 13.2566 ± 2.010 | .6264 ±.227 |
| day06 | 13.837 ±  2.42513 | 93.313 ±  2.025 | 71.658 ±  9.412 | 7.610 ±  3.683 | 10.489 ±  1.801 | .737 ±  .332 | 50.058 ±  13.811 | .075 ±  .034 | 12.0452 ± 2.517 | 10.845 ± 2.115 | .7396 ±.333 |
| day07 | 14.132 ±  3.262 | 94.921 ±  1.096 | 73.398 ±  9.393 | 8.182 ±  3.268 | 10.734 ±  2.035 | .564 ±  .148 | 49.876 ±  22.067 | .072 ±  .026 | 15.9571 ± 3.124 | 14.011 ± 3.386 | .7075 ±.3075 |
| day09 | 12.803 ±  2.769 | 94.292 ±  1.413 | 71.042 ±  10.945 | 7.727 ±  2.622 | 10.029 ±  1.901 | .5647 ±  .213 | 45.907 ±  14.994 | .081 ±  .018 | 13.5310 ± 3.463 | 12.567 ± 2.883 | .7698 ±.315 |
| day12 | 11.645 ±  3.028 | 94.683 ±  .893 | 71.583 ±  10.636 | 7.667 ±  2.737 | 9.887 ±  1.447 | .488 ±  .117 | 46.831 ±  7.706 | .079 ±  .018 | 13.050 ± 3.618 | 12.979 ± 3.498 | .5509 ±.247 |
| day14 | 12.964 ±  2.978 | 94.685 ±  .957 | 66.511 ±  6.575 | 7.707 ±  2.871 | 10.297 ±  1.670 | .545 ±  .206 | 49.80 ±  13.207 | .078 ±  .008 | 13.9643 ± 3.389 | 13.088 ± 2.795 | .7038 ±.342 |
| day19 | 14.498 ±  2.680 | 94.745 ±  1.376 | 71.198 ±  10.203 | 7.792 ±  2.382 | 10.414 ±  1.644 | .674 ±  .18422 | 45.73 ±  7.758 | .078 ±  .0264 | 15.7262 ± 3.148 | 13.786 ± 2.672 | .5660 ±.301 |
| day21 | 12.951 ±  2.1482 | 94.519 ±  1.295 | 70.751 ±  8.938 | 8.385 ±  2.775 | 9.888 ±  1.261 | .609 ±  .168 | 51.562 ±  18.493 | .082 ±  .02342 | 13.7714 ± 2.648 | 13.050 ± 2.258 | .5377 ±.315 |
| day24 | 14.190 ±  3.408 | 94.444 ±  1.091 | 63.215 ±  4.184 | 8.132 ±  1.994 | 10.249 ±  1.068 | .654 ±  .227 | 45.143 ±  6.206 | .088 ±  .0177 | 15.064 ± 4.048 | 13.905 ± 3.28 | .6528 ± .241 |
| day28 | 14.328 ±  3.783 | 94.451 ±  1.501 | 65.135 ±  4.659 | 7.654 ±  2.085 | 9.951 ±  1.323 | .731 ±  .229 | 42.864 ±  4.962 | .083 ±  .027 | 11.542 ± 2.176 | 11.041 ± 2.053 | .658 ± .243 |

Footnote: *p < 0.05, **p < 0.01, *p < 0.001.

Supplementary Table S2: Mean scores of each pulse waveform parameters for overall region (mean ± standard deviation) in vessel region (MV).

| MV | MBR | BOS | BOT | RR | FR | FAI | ATI | RI | MBR_max_ | MBR_min_ | AC |
| --- | --- | --- | --- | --- | --- | --- | --- | --- | --- | --- | --- |
| *P* value | *** | *** | *** |  | *** | *** |  | *** | *** | *** | *** |
| base | 39.445  ± 4.006 | 92.455  ± 1.354 | 74.993  ± 12.095 | 8.770  ±.770 | 10.544  ± ±.891 | 3.075  ±.641 | 44.445  ± 19.925 | .1168  ±.024 | 24.881 ±7.663 | 20.642  ± 5.684 | 2.964 ±.685 |
| day04 | 31.232  ± 6.026 | 95.028  ± 1.101 | 74.209  ± 15.780 | 8.388  ± 2.713 | 10.4451  ± .833 | 1.454  ±.545 | 50.935  ± 26.503 | .0772  .019 | 29.2591  ±3.070 | 29.250  ± 5.680 | 2.8151 ±.949 |
| day05 | 29.242  ± 8.448 | 94.515  ± 1.975 | 67.575  ± 7.308 | 8.811  ± 2.675 | 10.994  ± 2.152 | .975  ±.746 | 48.002  ± 8.182 | .0798  ±.034 | 29.209 ±7.272 | 27.159  ± 2.914 | 2.486 ±1.093 |
| day06 | 30.552  ± 10.108 | 94.801  ± 2.425 | 71.670  ± 9.152 | 7.978  ± 3.054 | 10.805  ± 2.144 | 1.847  ±.896 | 47.077  ± 15.668 | .0745  ±.038 | 25.006 ±4.923 | 23.464  ± 4.460 | 1.622 ±.808 |
| day07 | 31.972  ± 7.267 | 95.292  ± 1.329 | 72.129  ± 11.012 | 8.695  ± 2.482 | 10.912  ± 1.939 | 1.384  ±.487 | 49.752  ± 23.078 | .0677  ±.021 | 33.688 ±5.682 | 31.088  ± 5.504 | 2.201 ±.900 |
| day09 | 30.374  ± 7.980 | 94.594  ± 1.217 | 66.079  ± 4.621 | 8.132  ± 2.166 | 9.989  ± 1.617 | 1.356  ±.503 | 46.188  ± 13.480 | .0821  ±.020 | 28.281 ±8.055 | 25.664  ± 7.109 | 2.405 ±.987 |
| day12 | 29.892  ± 7.901 | 94.401  ± 1.345 | 66.554  ± 6.412 | 8.475  ± 2.563 | 9.819  ± 1.868 | 1.250  ±.490 | 47.932  ± 16.375 | .0817  ±.0209 | 26.786 ±7.680 | 24.064  ± 6.628 | 2.124 ±.636 |
| day14 | 28.778  ± 7.621 | 95.082  ± 1.428 | 68.513  ± 9.018 | 8.185  ± 1.974 | 10.063  ± 1.411 | 1.183  ± .521 | 49.322  ± 12.919 | .0717  ±.0201 | 28.050 ±6.665 | 26.154  ± 6.022 | 1.9906  ±.745 |
| day19 | 31.080  ± 7.708 | 95.086  ± 1.627 | 67.801  ± 6.635 | 8.445  ± 2.008 | 9.975  ± 1.391 | 1.560  ± .469 | 46.715  ± 11.655 | .0726  ±.0279 | 31.1250 ±6.474 | 28.716  ± 5.062 | 2.294 ±.949 |
| day21 | 30.960  ± 7.129 | 94.403  ± 1.399 | 68.285  ± 9.207 | 8.273  ± 2.399 | 9.754  ± 1.324 | 1.532  ±.812 | 47.547  ± 14.735 | .081  ±.022 | 29.504 ±6.987 | 27.226  ± 6.000 | 2.000 ±.638 |
| day24 | 33.449  ± 8.461 | 94.021  ±.932 | 64.540  ± 4.449 | 8.800  ± 2.281 | 10.073  ± 1.292 | 1.592  ±±.712 | 45.869  ± 7.909 | .089  ±.018 | 32.131 ±7.992 | 29.328  ± 7.512 | 2.262 ±.601 |
| day28 | 31.477  ± 11.604 | 94.884  ± 1.662 | 65.911  ± 5.444 | 8.002  ± 2.167 | 9.656  ± 1.503 | 1.577  .626 | 44.781  ± 6.731 | .074  ±.026 | 24.579 ±5.001 | 21.735  ± 3.592 | 1.935 ±.722 |

Footnote: *p < 0.05, **p < 0.01, *p < 0.001.

Supplementary Table S3: Mean scores of each pulse waveform parameters for overall region (mean ± standard deviation) in tissue region (MT).

| MT | MBR | BOS | BOT | RR | FR | FAI | ATI | RI | MBR_max_ | MBR_min_ | AC |
| --- | --- | --- | --- | --- | --- | --- | --- | --- | --- | --- | --- |
| *P* value | *** | *** | *** | 0.025 | *** | *** |  | *** | *** | *** | *** |
| base | 10.122  ± 1.249 | 91.700  ± 1.020 | 73.279  ± 12.028 | 8.701  ± 1.904 | 11.383  ± 1.373 | .6925  ±.122 | 41.720  ± 9.975 | .125  ± .023 | 7.547  ± 1.334 | 6.545 ±1.238 | .930 ±.132 |
| day04 | 9.267  ± 1.686 | 94.728  ± 1.894 | 72.279  ± 12.977 | 8.537  ± 3.615 | 11.366  ± 2.666 | .3340  ±.145 | 49.679  ± 15.979 | .077  ± .035 | 8.400  ± 1.265 | 7.3547  ±1.6728 | .5725  ±.248 |
| day05 | 9.259  ± 2.265 | 93.722  ± 1.622 | 72.235  ± 11.804 | 9.017  ± 2.963 | 11.286  ± 2.444 | .4396  ±.102 | 52.575  ± 21.739 | .094  ± .030 | 8.090  ± 1.842 | 7.8434  ±1.357 | .6500  ±.229 |
| day06 | 9.094  ± 2.546 | 93.788  ± 2.236 | 70.845  ± 10.696 | 7.228  ± 2.671 | 11.032  ± 2.014 | .5528  ±.291 | 47.249  ± 16.366 | .091  ± .034 | 7.494  ± 1.922 | 6.915 ±1.589 | .8350  ±.284 |
| day07 | 9.046  ± 2.038 | 94.688  ± 1.327 | 75.813  ± 7.978 | 8.215  ± 3.332 | 10.686  ± 1.820 | .4245  ± .151 | 48.117  ± 22.409 | .077  ± .024 | 9.435  ± 2.378 | 8.7038 ±2.171 | .7000  ±.186 |
| day09 | 8.289  ± 1.879 | 94.526  ± 1.357 | 71.122  ± 10.912 | 7.530  ± 2.901 | 10.501  ± 1.962 | .345  ± .099 | 46.303  ± 17.135 | .080  ± .017 | 8.241  ± 1.918 | 7.5340  ±1.768 | .7850  ±.348 |
| day12 | 8.423  ± 2.038 | 94.549  ± 1.125 | 72.990  ± 10.075 | 7.945  ± 2.603 | 10.013  ± 1.667 | .345  ± .097 | 50.766  ± 18.495 | .0757  ± .021 | 8.7887  ± 2.444 | 8.075 ±2.183 | .610 ±.228 |
| day14 | 8.371  ± 1.628 | 94.775  ± 1.161 | 67.726  ± 8.440 | 7.894  ± 1.805 | 9.975  ± 1.044 | .371  ±.111 | 48.128  ± 14.592 | .077  ±.019 | 8.571  ± 1.879 | 7.935 ±1.735 | .620 ±.318 |
| day19 | 8.800  ± 1.467 | 94.118  ± 1.172 | 70.943  ± 9.899 | 8.162  ± 1.634 | 10.211  ± 1.081 | .443  ±.088 | 48.139  ± 17.574 | .0840  ± .024 | 9.017  ± 1.767 | 8.401  ±1.416 | .600  ±.300 |
| day21 | 8.707  ± 1.695 | 94.201  ± 1.337 | 72.147  ± 10.086 | 8.022  ± 3.142 | 9.675  ± 1.493 | .428  ±.157 | 53.283  ± 18.894 | .0817  ± .023 | 8.864  ± 1.947 | 8.145 ±1.687 | .5475  ±.279 |
| day24 | 8.919  ± 2.008 | 94.311  ± 1.183 | 64.607  ± 6.881 | 7.320  ± 1.538 | 9.890  ±.611 | .441  ±.165 | 44.339  ± 13.420 | .0842  ± .017 | 9.150  ± 2.322 | 8.352 ±2.068 | .703  ±.209 |
| day28 | 8.862  ± 2.864 | 93.879  ± 1.196 | 63.877  ± 4.769 | 8.032  ± 2.150 | 9.600  ± 1.046 | .492  ±.165 | 47.713  ± 13.782 | .092  ± .019 | 8.067  ± 1.118 | 7.081 ±1.024 | .685  ±.229 |

Footnote: *p < 0.05, **p < 0.01, *p < 0.001.

Supplementary Table S4: Summary of p value for AC, S1 and S2 area for each region respectively.

|  | MA | | | MV | | | MT | | |
| --- | --- | --- | --- | --- | --- | --- | --- | --- | --- |
|  | S1_RR | Sall_FR | S2_FR | S1_RR | Sall_FR | S2_FR | S1_RR | Sall_FR | S2_FR |
| Base | 2.55 ± .023 | 4.58 ± .028 | 2.03 ± .266 | 2.93 ± .131 | 4.86 ± .199 | 2.08 ± .299 | 2.66 ± .028 | 4.91 ± .086 | 2.22 ± .306 |
| Day 14 | 3.97 ±.011 | 3.98 ± .004 | 1.64 ± .299 | 3.76 ± .061 | 3.83 ± .028 | 1.53 ± .215 | 3.74 ± .201 | 3.88 ± .119 | 1.71 ± .357 |
| Day 28 | 3.88 ±.16 | 3.91 ± .119 | 1.52 ± .216 | 3.87 ± .034 | 3.88 ± 0.27 | 1.44 ± .071 | 3.47 ± .071 | 3.91 ± .209 | 1.60 ± .311 |


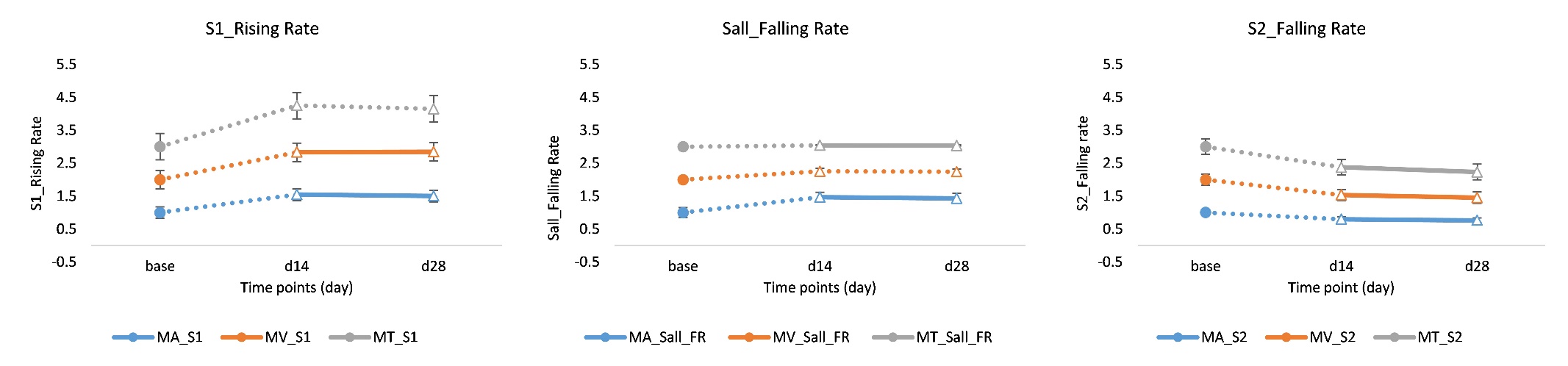


Supplementary Figure. 1 (A-C). Longitudinal changes in waveform area at different days. The line graph represents area of, A): S1 area of Rising Rate, B): Sall area of Falling Rate and C): S2 area of Falling Rate, for each region respectively. Vertical bars represent the standard error of means. Results from one-way repeated measures ANOVA are display in the line graphs, dotted line represent significant difference between each time points. Symbols represent statistically significant difference between base with respective time points (empty circles: *p* < 0.05 and empty triangle: *p* < 0.001).
